# Newcastle-Ottawa Scale: comparing reviewers’ to authors’ assessments

**DOI:** 10.1186/1471-2288-14-45

**Published:** 2014-04-01

**Authors:** Carson Ka-Lok Lo, Dominik Mertz, Mark Loeb

**Affiliations:** 1 Department of Medicine, University of Toronto, Toronto Ontario, Canada; 2 Department of Medicine, McMaster University, Hamilton Ontario, Canada; 3 Department of Clinical Epidemiology and Biostatistics, McMaster University, Hamilton Ontario, Canada; 4 Michael G. DeGroote Institute for Infectious Diseases Research, McMaster University, Hamilton Ontario, Canada; 5 Department of Pathology and Molecular Medicine, McMaster University, MDCL 3203, 1200 Main St W, Hamilton, Ontario L8N 3Z5, Canada

**Keywords:** Newcastle Ottawa Scale, Inter-rater, Reliability, Validity, Risk of bias, Observational studies

## Abstract

**Background:**

Lack of appropriate reporting of methodological details has previously been shown to distort risk of bias assessments in randomized controlled trials. The same might be true for observational studies. The goal of this study was to compare the Newcastle-Ottawa Scale (NOS) assessment for risk of bias between reviewers and authors of cohort studies included in a published systematic review on risk factors for severe outcomes in patients infected with influenza.

**Methods:**

Cohort studies included in the systematic review and published between 2008–2011 were included. The corresponding or first authors completed a survey covering all NOS items. Results were compared with the NOS assessment applied by reviewers of the systematic review. Inter-rater reliability was calculated using kappa (*K*) statistics.

**Results:**

Authors of 65/182 (36%) studies completed the survey. The overall NOS score was significantly higher (p < 0.001) in the reviewers’ assessment (median = 6; interquartile range [IQR] 6–6) compared with those by authors (median = 5, IQR 4–6). Inter-rater reliability by item ranged from slight (*K* = 0.15, 95% confidence interval [CI] = −0.19, 0.48) to poor (*K* = −0.06, 95% CI = −0.22, 0.10). Reliability for the overall score was poor (*K* = −0.004, 95% CI = −0.11, 0.11).

**Conclusions:**

Differences in assessment and low agreement between reviewers and authors suggest the need to contact authors for information not published in studies when applying the NOS in systematic reviews.

## Background

One of the most important aspects of systematic reviews is the assessment of the risk of bias of included studies [1]. Evaluating the reliability and validity of risk of bias assessment scores can help generate better assessment tools. The Newcastle-Ottawa Scale (NOS) is a risk of bias assessment tool for observational studies that is recommended by the Cochrane Collaboration [1,2]. Some characteristics of this tool have been assessed, such as inter-rater reliability, which has been variable ranging from fair to high depending on the types of observational studies assessed and the raters [3,4]. In addition to the potentially limited inter-rater reliability, lack of reporting of methodological details in published articles may potentially distort the risk of bias assessment as it was shown for randomized controlled trials (RCT) [5,6].

The objectives of our study were therefore to compare the NOS assessment between reviewers with access to the publications only and the authors of published studies, using the list of studies included in a recently published systematic review of observational studies on risk factors to predict severe outcomes in patients with influenza [7]. We hypothesized that reviewers in the systematic review who had to rely on the published information would have assigned lower NOS scores (i.e., greater risk of bias) compared with its authors, and that the agreement between authors and reviewers would be marginal.

## Methods

### Eligibility criteria

Authors of studies included in a published systematic review on risk factors for severe outcomes in patients with influenza and published between 2008 and 2011 were included [7]. More remote publications were not considered, as the contact information was more likely no longer accurate and authors may not recall details of their studies.

### Quality assessment: NOS and NOS-derived survey

The NOS assigns up to a maximum of nine points for the least risk of bias in three domains: 1) selection of study groups (four points); 2) comparability of groups (two points); and 3) ascertainment of exposure and outcomes (three points) for case–control and cohort studies, respectively (Additional file 1) [2]. Survey questions were developed based on the NOS’ questions covering all three domains so that authors could provide detailed information about their studies. Because the NOS questionnaires are slightly different between case–control and cohort studies, two surveys were created.

Vaccination status for influenza and antiviral treatment were defined as the most important covariates that defined comparability, consistent with the NOS assessment conducted by the reviewers in the systematic review [7].

The corresponding author and the first author were contacted by email and were provided with the link to the actual electronic survey. An online survey was provided to all authors where their name, responses and timestamp were securely documented. Authors were required to respond to all questions provided prior to submission and answered follow-up questions only when prompted to. An example of a follow-up question: For *ascertainment of exposure*, authors who claimed using structured interviews had to also report whether the interviewers were blinded to exposure status. A feedback form was provided at the end of the survey for authors to comment on the survey. A total of two reminders were sent to authors not completing the survey.

### Analysis

Questions in the survey were matched to the original NOS items, so that points were awarded to the respective answers.

Differences in the NOS scores between the reviewers’ and authors’ assessment were compared with the Wilcoxon paired sign-rank test.

Inter-rater agreement Cohen’s kappa was used to compare agreement between the reviewers from the systematic review and the authors’ assessments. The scores were compared between raters across each NOS domain or item. Weighted kappa was performed for variables with more than two classifications (i.e., comparability had scores of 0, 1, and 2). A weighted kappa was also performed for the total NOS scores.

Agreement interpretation was based on established categorizations: poor (*K* < 0.00), slight (0.00-0.20), fair (0.21-0.40), moderate (0.41-0.60), substantial (0.61-0.80), and almost perfect (0.81-1.00) [8].

MedCalc® was used for all weighted/non-weighted kappa analyses [9,10] and for the Wilcoxon paired sign-rank test. Mean and standard deviation (SD), median and interquartile range (IQR) were reported.

## Results

Of the 239 studies included in the systematic review, 196 studies (82%; 6 case–control and 190 cohort studies) met the inclusion criteria for this study (i.e., published from 2008 to 2011). Six cohort studies were excluded, as there was no information on contacting authors (three articles published by the World Health Organization (WHO) and three articles by Centre for Disease Control and Prevention (CDC)). An additional two studies were excluded, as contacting authors could not be identified.

Authors of 65/182 (36%) eligible cohort studies and 2/6 (33%) case–control studies completed the survey (Additional files 2 and 3). Given the sparse data on case–control studies, these studies were excluded from analysis.

Reviewer assessment was similar for included (n = 65, mean NOS score = 5.85, median = 6, IQR 6–6) and excluded studies (n = 117, mean = 5.81, median = 6, IQR 5–6).

**Figure 1:**
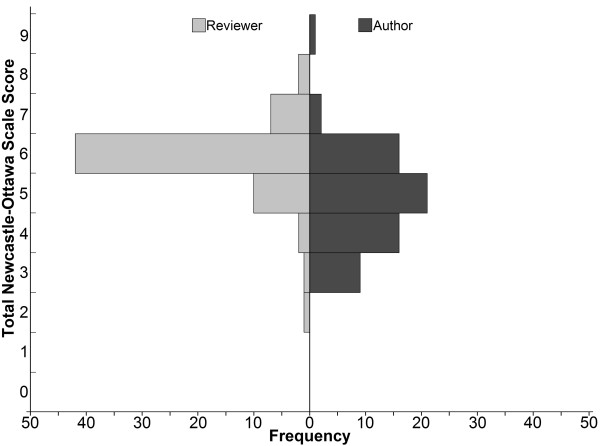
**Distribution of total scores for the Newcastle-Ottawa Scale (NOS) for cohort studies.** Reviewers (left) and authors (right) evaluated for risk of bias for cohort studies (n = 65).

**Figure 2:**
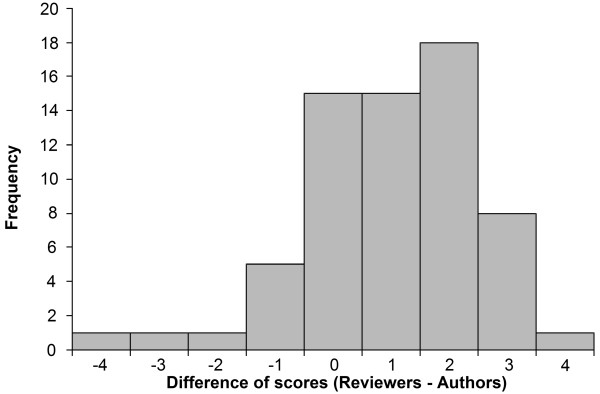
**Differences in Newcastle-Ottawa Scale (NOS) total score between reviewers and authors.** The total score assigned for each cohort study by reviewers was subtracted with the total score assigned by authors.

The distribution of NOS scores by reviewers and authors for cohort studies included in this analysis is shown in Figure 1. The risk of bias by the study authors was felt to be higher, with a lower average NOS score (mean = 4.85, SD = 1.19, median = 5, IQR 4–6) compared with the NOS reviewers (mean = 5.85, SD = 0.94, median = 6, IQR 6–6). The difference was statistically significant (p < 0.001, Wilcoxon paired signed-rank test). Thirty (46%) out of the 65 cohort studies had overall NOS scores that were two or more points apart, of which 27 studies had reviewers scoring two or more points higher (i.e., lower risk of bias) than authors (Figure 2). Eight and one of these 30 studies reviewers scored three and four points higher than authors, respectively.

Inter-rater reliability for the 65 cohort studies across the NOS items is presented in Table 1. Reliability was slight (i.e., *K* of 0.00 to 0.15) for the majority of items (*representativeness of the exposed cohort*, *selection of the non-exposed cohort*, *demonstration that outcome of interest was not present at the start of study*, *comparability of cohorts*, and *adequacy of follow-up of cohorts*). The items a*scertainment of exposure*, *assessment of outcome*, and “*was follow-up long enough for outcomes to occur*” had poor reliability (i.e., *K* of −0.06 to −0.02). Reliability for the total NOS score was poor (*K* = −0.004, 95% confidence interval [CI] = −0.11, 0.11).

**Table 1:** Inter-rater reliability on the Newcastle-Ottawa Scale (NOS) assessments, by item

**Item**	**Agreement *K *** **(95% CI)**	**Interpretation **[8]	**0 point difference**^**c**^	**±1 point difference**^**c**^	**> ±2 points difference**^**c**^
Representativeness of the exposed cohort	0.03 (−0.10, 0.15)	Slight	43 (66.2%)	22 (33.8%)	0 (0%)
Selection of the non-exposed cohort	0.00 (0.00, 0.00)	Slight	53 (81.5%)	12 (18.5%)	0 (0%)
Ascertainment of exposure	−0.02 (−0.08, 0.04)	Poor	12 (18.5%)	53 (81.5%)	0 (0%)
Demonstration that outcome of interest was not present at start of study	0.09 (−0.16, 0.35)	Slight	47 (72.3%)	18 (27.7%)	0 (0%)
Comparability	0.00^a^ (−0.11, 0.12)	Slight	38 (58.5%)	18 (27.7%)	9 (13.8%)
Assessment of outcome	−0.04 (−0.09, 0.00)	Poor	59 (90.8%)	6 (9.2%)	0 (0%)
Was follow-up long enough for outcomes to occur	−0.06 (−0.22, 0.10)	Poor	31 (47.7%)	34 (52.3%)	0 (0%)
Adequacy of follow-up of cohorts	0.15 (−0.19, 0.48)	Slight	57 (87.7%)	8 (12.3%)	0 (0%)
Total NOS score	−0.004^a^ (−0.11, 0.11)	Poor	15 (23.1%)	20 (30.8%)	30 (46.1%)
Total categorized NOS score	0.14^b^ (−0.02, 0.29)	Slight	44 (67.7%)	21 (32.3%)	0 (0%)

*Abbreviation*: 95% CI = 95% confidence interval.

^a^Linear weighted kappa was used for both *Comparability* and *Total NOS score*; other kappas were not weighted (i.e., Cohen’s kappa was applied).

^b^Quadratic weighted kappa was used assuming the difference between very high risk, high risk and low risk were comparably unequal.

^c^Number of studies with a 0, ±1, or more than ±2 points difference between reviewer and author assessments, separated by item.

A second inter-rater reliability test was performed using weighted kappa (*K*) comparing total NOS scores categorized into three groups: very high risk of bias (0 to 3 NOS points), high risk of bias (4 to 6), and low risk of bias (7 to 9). Quadratic kappa was applied because the groups “very high risk” vs. “high risk” and “high risk” vs. “low risk” were treated as comparably different. Reliability for the categorized total NOS score was slight (*K* = 0.14, 95% CI = −0.02, 0.29; Table 1). Compared with the poor agreement from the initial inter-rater reliability test, the categorized NOS score showed better but still only slight agreement.

## Discussion

By comparing overall scores and inter-rater reliability of the NOS quality assessment tool between reviewers and authors, we found remarkably low inter-rater reliability. The majority of the cohort studies were rated as being at higher risk of bias by authors than by reviewers. That is, the reviewers assigned higher NOS scores by two or more points of a total of nine points for the overall NOS score.

Inter-rater reliability between authors and reviewers was remarkably low and the agreement across items was only minimal. The item about *adequacy of follow-up of cohorts* had the highest kappa value of 0.15 (95% CI = −0.19, 0.48), despite being considered only as slight agreement. The comparably small room for subjectivity might explain the relatively high kappa value given that the authors simply had to indicate whether or not there was loss to follow-up for their cohort, and whether it would potentially have introduced bias into their study. The overall lack of agreement between reviewers and authors persisted even after performing a second inter-rater reliability test when categorizing into three groups of risk of bias. Of note, the difference in NOS scores was 2 or higher in almost half of the studies. While one may argue that a one point difference would not have any practical implications, a two point difference would likely be regarded as clinically important given that this would reflect a >20% (i.e., 2/9 points) difference in the assessment.

One possible explanation for these differences is that the reviewers may not have had all information needed available from the published article in order to assess the risk of bias reliably. For example, when evaluating *representativeness of exposed cohort* reviewers may treat the study population as truly representative, but the author knew that the study group was not representative for the average population in the respective setting.

Methodological studies have found that reviewers may overestimate the risk of bias of randomized controlled trials due to unclear or lacking details from insufficient reporting by authors [5,6]. In one of these studies involving 98 RCTs, 55% failed to report allocation concealment and from 25% to 96% did not report blinding status (e.g., for participants, data analysts) [5]. However, once authors were contacted, it was noted that appropriate concealment and blinding were in fact in place in many of these studies [5]. In other words, reviewers without the unpublished details from authors would have overestimated the risk of bias for RCTs, contrary to our findings when using NOS for observational studies. One possible explanation might be that NOS allows for more subjectivity than when assessing RCTs; lack of information in RCTs automatically results in higher risk of bias, whereas NOS requires the reviewer to decide subjectively on the risk of bias for each item based on the information available in the report. Others have also found the NOS tool’s decision rules and guidelines to be vague and difficult to use [3]. For example, the difference between a “structured interview” and “written self-report” was difficult to determine if the study used a structured validated survey that was completed independently by study participants [3]. Similar ambiguity was found in our investigation for the item *ascertainment of exposure,* where NOS reviewers identified 59 of the 65 cohort studies using secure records (i.e., one point assigned). In contrast, 44 out of the 65 authors claimed to be using medical records (i.e., no points assigned).

### Inter-rater reliability

The developers of the NOS evaluated the tool for face and criterion validity by comparing it with an established scale by Downs and Black [11]. Ten cohort and 10 case–control studies about the association between breast cancer and hormone replacement therapy were examined. Criterion validity showed strong and moderate agreement for cohort (intra-class correlation coefficient [ICC] = 0.88) and case–control studies (ICC = 0.62), respectively. Inter-rater reliability was also high for both cohort (ICC = 0.94) and case–control studies (ICC = 0.82).

To our knowledge, only two other studies have examined inter-rater reliability of NOS other than those conducted by the developers of the tool [3,4]. Contrary to findings from developers of the tool, other studies have found overall low inter-rater reliability. In a study by Hartling [3], two reviewers applied NOS to 131 cohort studies included in eight meta-analyses on different medical topics. Inter-rater reliability between reviewers ranged from poor (*K* = −0.06, 95% CI = −0.20, 0.07) to substantial (*K* = 0.68, 95% CI = 0.47, 0.89), although eight out of nine of the NOS items had *K* values <0.50 [3]. Reliability for overall NOS score was fair (*K* = 0.29, 95% CI = 0.10, 0.47). A similar lack of agreement was found in a study applying the NOS to observational studies on cognitive impairment following electroconvulsive therapy for major depressive disorders [4]. Using inexperienced student raters, inter-rater reliability for cohort studies ranged from *K* = −0.14 (95% CI = −0.28, 0.00) to *K* = 0.39 (95% CI = −0.02, 0.81), and for case–control studies from *K* = −0.20 (95% CI = −0.49, 0.09) to *K* = 1.00 (95% CI = 1.00, 1.00). All nine of the NOS items and seven out of nine items had *K* values <0.50 for cohort and case–control studies, respectively. However, the findings of the latter study may also explain to some extent the disagreement between methodically trained reviewers and authors of study: In our study, the disagreement between raters may also have been attributable to the authors’ lack of experience applying the NOS.

### Limitations

One limitation of our study was the relatively small sample size of 65 cohort studies. With a response rate of 36%, selection bias may be present as authors who responded to the survey might have a different level of expertise and interest in research methodology than non-responders. Also, all studies included were from one field of medicine. Notably however, the reviewer assessments of the risk of bias were similar for the included and excluded studies. It remains unclear whether the difference between the reviewer and author’s assessments were due to different information available to them for assessment, or whether authors’ lack of familiarity with the NOS tool resulted in this finding. Given that the one item (i.e., *representativeness of the exposed cohort*) that would not need to be downgraded based on lack of information was similarly low in agreement as items that would expect downgrading for lack of reporting, subjective interpretation was probably the primary driver resulting in the low agreement between reviewers and authors. This would emphasize, as suggested by Hartling [3] and Oremus [4], that training and detailed guidance is needed in order to appropriately apply the NOS. Although the survey explained in detail how the assessment should be conducted, lack of familiarity with the NOS tool by the authors may have negatively affected their performance.

Another limitation is the nature of the inter-rater reliability test, that is, some have considered kappa to be an overly conservative measure of agreement [12]. Since the kappa value depends on the observed categories’ frequencies, the test might underestimate agreement for a category. An example was the item *selection of the non-exposed cohort*, where 82% of the time both reviewers and authors agreed that the non-exposed were derived from the same population as the exposed (i.e., one point assigned). However, there were no occurrences where both raters agreed that the non-exposed were not derived from the same population as the exposed, which resulted in an underestimation of the agreement. A larger sample of studies may have circumvented this problem.

## Conclusions

It remains unclear whether the difference between the reviewer and authors’ assessment and low inter-rater reliability was due to lack of information available to reviewers in published influenza papers, or whether it was the authors’ lack of familiarity with the NOS items. However, it is prudent to conclude that systematic reviewers should contact authors for information not published in the study to supplement the use of the risk of bias assessment tool. The room for subjectivity in the NOS tool may have negatively affected inter-rater reliability. Revised or new instruments using less subjective items may improve inter-rater reliability and potentially validity of the risk of bias assessment of observational studies in systematic review.

## Abbreviations

NOS: Newcastle-Ottawa Scale; RCT: Randomized controlled trial; SD: Standard deviation; IQR: Interquartile range; WHO: World Health Organization; CDC: Centre for Disease Control and Prevention; CI: Confidence interval; ICC: Intra-class correlation coefficient.

## Competing interests

The authors declare that they have no competing interests.

## Authors’ contributions

DM and ML designed the study. CKL drafted the survey, all authors contributed to revising the survey and provided a final version to be sent to authors of interest. DM responded to all inquiries from authors regarding the survey. CKL contributed to data collection, data analysis, and interpretation. DM and ML provided feedback on interpretation of data. CKL drafted the manuscript. All authors critically revised the manuscript and provided final approval of the version to be published.

## Pre-publication history

The pre-publication history for this paper can be accessed here:

http://www.biomedcentral.com/1471-2288/14/45/prepub

## Supplementary Material

Additional file 1 **Original NOS and survey questions for cohort studies.** Original NOS (left) and survey questions (right) that were sent to authors. Criteria for awarding points in the survey were matched with the original NOS, as indicated by corresponding symbols in the document. Click here for file

Additional file 2 **Cohort studies included in analysis.** References of all 65 cohort studies who completed the survey and included in data analysis. Click here for file

Additional file 3 **Raw data for NOS reviewer and author assessments.** Risk of bias assessments of all 65 cohort studies by NOS reviewers and authors. Scores were tabulated and separated by NOS domain and item, with total scores included. Click here for file
