# Supplementary material for: Newcastle-Ottawa Scale: comparing reviewers’ to authors’ assessments
Source: BMC Med Res Methodol. 2014 Apr 1;14:45. doi: 10.1186/1471-2288-14-45 (PMC4021422; doi:10.1186/1471-2288-14-45)
Supplement: Additional file 1 — Original NOS and survey questions for cohort studies. Original NOS (left) and survey questions (right) that were sent to authors. Criteria for awarding points in the survey were matched with the original NOS, as indicated by corresponding symbols in the document. [file 1471-2288-14-45-S1.pdf]

Additional file 1. Original NOS and survey questions for cohort studies

Original NOS items [2]	Matched survey questions
Selection	
a) Representativeness of the exposed cohort<br>- truly representative of the average ________ (describe) in the community†<br>- somewhat representative of the average ________ in the community†<br>- selected group of users e.g., nurses, volunteers	Were ALL eligible, exposed individuals (i.e., those with risk factors) from a defined community/hospital population during a given time period enrolled?<br>- yes†<br>- no
b) Selection of the non-exposed cohort<br>- drawn from the same community as the exposed cohort†<br>- drawn from a different source<br>- no description of the derivation of the non-exposed cohort	Were non-exposed (patients without risk factors) derived from the same population as the exposed?<br>- yes†<br>- no
c) Ascertainment of exposure<br>- secure record (e.g., surgical records)†<br>- structured interview†<br>- written self report<br>- no description	Please specify how the presence of risk factors were ascertained:<br>- secure record (e.g., surgical records)†<br>- structured interviews<br>*- were interviewers blinded to exposure status (yes†/no)*<br>- written self reports<br>- medical records<br>- other (please specify): ________
d) Demonstration that outcome of interest was not present at start of study<br>- yes†<br>- no	Were the outcomes (e.g., pneumonia, hospital- or ICU admission, ventilator support) of interest already present at enrollment of the patients?<br>- yes<br>- no†

Comparability

e) Comparability of cohorts on the basis of the design or analysis	We considered vaccination status and antiviral treatment as the two single most important confounding factors that could prevent severe outcomes in patients with influenza.<br><br>Have you either matched or adjusted for these factors in your analysis?
- study controls for ________ (select most important factor)[†]<br>- study controls for any additional factor[†] (This criteria could be modified to indicate specific control for a second important factor)	- matched/adjusted for both factors (vaccination status and antiviral treatment)[††]<br>-matched/adjusted for only one of these factors[†]<br>- not matched/adjusted for these factors, but matched/adjusted for other factors.<br>- *If so, which factors?*[§]<br>- no matching or adjustment at all

Outcome

f) Assessment of outcome<br>- independent blind assessment[†]<br>- record linkage[†]<br>- self report<br>- no description	How were outcomes ascertained?<br>- >1 person assessing patients independently[‡]<br>- medical/hospital records as the primary source[‡]<br>- record linkage (e.g., ICD codes in database)[‡]<br>- self report<br>- other, please specify: ________<br><br>*Were the assessors blinded to risk factor status?*<br>- *yes*[‡]<br>- *no*
g) Was follow-up long enough for outcomes to occur?<br>- yes (select an adequate follow up period for outcome of interest)[†]<br>- no	What was the length of time chosen for follow-up for outcomes to occur: ________[Δ]
h) Adequacy of follow up of cohorts<br>- complete follow up - all subjects accounted for[†]<br>- subject lost to follow up unlikely to introduce bias: small number lost > ___%	Did you have loss to follow up, i.e., outcome data not available?<br>- yes<br>- no[†]<br><br>- *If yes, what was the percentage of loss to follow-up:* ________________

(select an adequate %) follow up, or description provided of those lost†<br>- follow up rate < ___% (select an adequate %) and no description of those lost<br>- no statement	- *If yes, have you compared the baseline data of those with loss to follow-up in comparison to those included and did the prevalence of risk factors differ?*<br>- *yes*<br>- *no†*

Abbreviations: NOS = Newcastle-Ottawa Scale; ICU = intensive care unit; ICD = international classification of diseases

† one point awarded; †† two points awarded; § conditional point awarded upon agreement by all authors; ‡ one point awarded if answer chosen for both *How were outcomes ascertained?* and *Were the assessors blinded to risk factor status?* were indicated by symbol (‡); ∆ one point awarded if follow up period was adequate (i.e., the duration of hospitalization or until discharge/death); italics indicated follow-up and conditional questions given only to authors who responded specific answers in the survey
