# Supplementary material for: Newcastle-Ottawa Scale: comparing reviewers’ to authors’ assessments
Source: BMC Med Res Methodol. 2014 Apr 1;14:45. doi: 10.1186/1471-2288-14-45 (PMC4021422; doi:10.1186/1471-2288-14-45)
Supplement: Additional file 2 — Cohort studies included in analysis. References of all 65 cohort studies who completed the survey and included in data analysis. [file 1471-2288-14-45-S2.pdf]

Additional file 2. Cohort studies included in analysis

1. Al-Khuwaitir TS, Al-Abdulkarim AS, Abba AA, Yousef AM, El-Din MA, Rahman KT, Ali MA, Mohamed ME, Arnous NE: **H1N1 influenza A. Preliminary evaluation in hospitalized patients in a secondary care facility in Saudi Arabia.** *Saudi Med J* 2009, **30:**1532-1536.
2. Allard R, Leclerc P, Tremblay C, Tannenbaum TN: **Diabetes and the severity of pandemic influenza A (H1N1) infection.** *Diabetes Care* 2010, **33:**1491-1493.
3. Bagdure D, Curtis DJ, Dobyns E, Glodé MP, Dominguez SR: **Hospitalized children with 2009 pandemic influenza A (H1N1): comparison to seasonal influenza and risk factors for admission to the ICU.** *PLoS ONE [Electronic Resource]* 2010, **5:**e15173.
4. Bantar C, Oliva ME, Ré HA, Sandillú M, Franco D, Izaguirre M, Carmagnac A, Vesco E, Grieve CG, Duarte J, Basso P: **Severe acute respiratory disease in the setting of an epidemic of swine-origin type A H1N1 influenza at a reference hospital in Entre Rios, Argentina.** *Clin Infect Dis* 2009, **49:**1458-1460.
5. Belongia EA, Irving SA, Waring SC, Coleman LA, Meece JK, Vandermause M, Lindstrom S, Kempf D, Shay DK: **Clinical characteristics and 30-day outcomes for influenza A 2009 (H1N1), 2008-2009 (H1N1), and 2007-2008 (H3N2) infections.** *JAMA* 2010, **304:**1091-1098.
6. Bettinger JA, Sauvé LJ, Scheifele DW, Moore D, Vaudry W, Tran D, Halperin SA, Pelletier L: **Pandemic influenza in Canadian children: a summary of hospitalized pediatric cases.** *Vaccine* 2010, **28:**3180-3184.
7. Caprotta G, González Crotti P, Primucci Y, Alesio H, Esen A: **Influenza A H1N1 respiratory infection in an intensive care unit in Argentina. [Spanish] Infeccion respiratoria por influenza A H1N1 en cuidados intensivos de la Republica Argentina.** *Anales de Pediatría* 2010, **72:**62-66.
8. Chien JMF, Tan BH, Yang KS, Tan TT, Low CY, Kurup A, Leong HN, Low JGH, Kang ML, Chlebicki MP, Koh YL: **Severe infection with H1N1 requiring intensive care - lessons for preparedness programmes.** *Ann Acad Med Singapore* 2010, **39:**328-332.
9. Chien Y-S, Su C-P, Tsai H-T, Huang AS, Lien C-E, Hung M-N, Chuang J-H, Kuo H-S, Chang S-C: **Predictors and outcomes of respiratory failure among hospitalized pneumonia patients with 2009 H1N1 influenza in Taiwan.** *J Infect* 2010, **60:**168-174.
10. Choi WI, Yim JJ, Park J, Kim SC, Na MJ, Lee WY, Hong SB, Choi HS, Jang SH, Kim WJ, Jeon K, Kim JH, Choi JC, Lee CH, Kim CH, Kim JY: **Clinical characteristics and outcomes of H1N1-associated pneumonia among adults in South Korea.** *Int J Tuberc Lung Dis* 2011, **15:**270-275.
11. Chudasama RK, Patel UV, Verma PB: **Hospitalizations associated with 2009 influenza A (H1N1) and seasonal influenza in Saurashtra region, India.** *J Infect Dev Ctries* 2010, **4:**834-841.
12. Creanga AA, Johnson TF, Graitcer SB, Hartman LK, Al-Samarrai T, Schwarz AG, Chu SY, Sackoff JE, Jamieson DJ, Fine AD, Shapiro-Mendoza CK, Jones LE, Uyeki TM, Balter S, Bish CL, Finelli L, Honein MA: **Severity of 2009 pandemic influenza A (H1N1) virus infection in pregnant women.** *Obstet Gynecol* 2010, **115:**717-726.
13. Cui W, Zhao H, Lu X, Wen Y, Zhou Y, Deng B, Wang Y, Wang W, Kang J, Liu P: **Factors associated with death in hospitalized pneumonia patients with 2009 H1N1 influenza in Shenyang, China.** *BMC Infect Dis* 2010, **10:**145.

14. Di Giambenedetto S, Zileri Dal Verme L, Sali M, Farina S, Di Cristo V, Manzara S, De Luca A, Pignataro G, Prosperi M, Di Franco A, Gentiloni Silveri N, Delogu G, Cauda R, Fabbiani M, Fadda G: **Clinical presentation, microbiological features and correlates of disease severity of 2009 pandemic influenza A (H1N1) infection.** *Eur J Clin Microbiol Infect Dis* 2011, **30:**541-549.
15. Dominguez-Cherit G, Lapinsky SE, Macias AE, Pinto R, Espinosa-Perez L, de la Torre A, Poblano-Morales M, Baltazar-Torres JA, Bautista E, Martinez A, Martinez MA, Rivero E, Valdez R, Ruiz-Palacios G, Hernández M, Stewart TE, Fowler RA: **Critically Ill patients with 2009 influenza A(H1N1) in Mexico.** *JAMA* 2009, **302:**1880-1887.
16. D'Ortenzio E, Renault P, Jaffar-Bandjee MC, Gaüzère BA, Lagrange-Xélot M, Fouillet A, Poubeau P, Winer A, Bourde A, Staikowsky F, Morbidelli P, Rachou E, Thouillot F, Michault A, Filleul L: **A review of the dynamics and severity of the pandemic A(H1N1) influenza virus on Reunion Island, 2009. (Lessons from the H1N1 influenza pandemic in French overseas territories and interim reports from metropolitan France.).** *Clin Microbiol Infect* 2010, **16:**309-316.
17. Dubar G, Azria E, Tesnière A, Dupont H, Le Ray C, Baugnon T, Matheron S, Luton D, Richard J-C, Launay O, Tsatsaris V, Goffinet F, Mignon A: **French experience of 2009 A/H1N1v influenza in pregnant women.** *PLoS ONE [Electronic Resource]* 2010, **5**.
18. Echevarria-Zuno S, Mejía-Aranguré JM, Mar-Obeso AJ, Grajales-Muñiz C, Robles-Pérez E, González-Léon M, Ortega-Alvarez MC, Gonzalez-Bonilla C, Rascón-Pacheco RA, Borja-Aburto VH: **Infection and death from influenza A H1N1 virus in Mexico: a retrospective analysis.** *Lancet* 2009, **374:**2072-2079.
19. Eriksson C, Randolph A: **Factors associated with respiratory failure and mortality among children hospitalized with seasonal influenza and pandemic 2009 H1N1.** *Crit Care Med* 2010, **38:**A6.
20. Estenssoro E, Rios FG, Apezteguia C, Reina R, Neira J, Ceraso DH, Orlandi C, Valentini R, Tiribelli N, Brizuela M, Balasini C, Mare S, Domeniconi G, Ilutovich S, Gomez A, Giuliani J, Barrios C, Valdez P, Registry of the Argentinian Society of Intensive Care: **Pandemic 2009 influenza A in Argentina: a study of 337 patients on mechanical ventilation.** *Am J Respir Crit Care Med* 2010, **182:**41-48.
21. Fasina FO, Ifende VI, Ajibade AA: **Avian influenza A (H5N1) in humans: lessons from Egypt.** *Euro Surveill* 2010, **15:**19473.
22. Gérardin P, Amrani RE, Cyrille BA, Gabrièle M, Guillermin P, Boukerrou M, Boumahni B, Randrianaivo H, Winer A, Rouanet JF, Bohrer M, Jaffar-Bandjee MC, Robillard PY, Barau G, Michault A: **Low clinical burden of 2009 pandemic influenza A (H1N1) infection during pregnancy on the island of la reunion.** *PLoS ONE [Electronic Resource]* 2010, **5:**1-11.
23. Gooya MM, Soroush M, Mokhtari-Azad T, Haghdoost AA, Hemati P, Moghadami M, Sabayan B, Heydari ST, Razavi SHE, Lankarani KB: **Influenza a (H1N1) pandemic in Iran: Report of first confirmed cases from June to November 2009.** *Arch Iran Med* 2010, **13:**91-98.
24. Gordon CL, Johnson PDR, Permezel M, Holmes NE, Gutteridge G, McDonald CF, Eisen DP, Stewardson AJ, Edington J, Charles PGP, Crinis N, Black MJ, Torresi J, Grayson ML: **Association between severe pandemic 2009 influenza A (H1N1) virus infection and immunoglobulin G(2) subclass deficiency.** *Clin Infect Dis* 2010, **50:**672-678.

25. Gubbels S, Perner A, Valentiner-Branth P, Molbak K: **National surveillance of pandemic influenza A(H1N1) infection-related admissions to intensive care units during the 2009-10 winter peak in Denmark: two complementary approaches.** *Euro Surveill* 2010, **15**.
26. Heltzer ML, Coffin SE, Maurer K, Bagashev A, Zhang Z, Orange JS, Sullivan KE: **Immune dysregulation in severe influenza.** *J Leukoc Biol* 2009, **85:**1036-1043.
27. Henzler T, Meyer M, Kalenka A, Alb M, Schmid-Bindert G, Bartling S, Schoepf JU, Schoenberg SO, Fink C: **Image Findings of Patients with H1N1 Virus Pneumonia and Acute Respiratory Failure.** *Acad Radiol* 2010, **17:**681-685.
28. Jiménez MF, El Beitune P, Salcedo MP, Von Ameln AV, Mastalir FP, Braun LD: **Outcomes for pregnant women infected with the influenza A (H1N1) virus during the 2009 pandemic in Porto Alegre, Brazil.** *Int J Gynaecol Obstet* 2010, **111:**217-219.
29. Jouvet P, Hutchison J, Pinto R, Menon K, Rodin R, Choong K, Kesselman M, Veroukis S, Andre Dugas M, Santschi M, Guerguerian A-M, Withington D, Alsaati B, Joffe AR, Drews T, Skippen P, Rolland E, Kumar A, Fowler R: **Critical illness in children with influenza A/pH1N1 2009 infection in Canada.** *Pediatr Crit Care Med* 2010, **11:**603-609.
30. Kelly PM, Kotsimbos T, Reynolds A, Wood-Baker R, Hancox B, Brown SGA, Holmes M, Simpson G, Bowler S, Waterer G, Irving LB, Jenkins C, Thompson PJ, Cheng AC: **FluCAN 2009: initial results from sentinel surveillance for adult influenza and pneumonia in eight Australian hospitals.** *Med J Aust* 2011, **194:**169-174.
31. Kumar A, Zarychanski R, Pinto R, Cook DJ, Marshall J, Lacroix J, Stelfox T, Bagshaw S, Choong K, Lamontagne F, Turgeon AF, Lapinsky S, Ahern SP, Smith O, Siddiqui F, Jouvet P, Khwaja K, McIntyre L, Menon K, Hutchison J, Hornstein D, Joffe A, Lauzier F, Singh J, Karachi T, Wiebe K, Olafson K, Ramsey C, Sharma S, Dodek P, et al: **Critically ill patients with 2009 influenza A(H1N1) infection in Canada.** *JAMA* 2009, **302:**1872-1879.
32. Kute VB, Godara SM, Goplani KR, Gumber MR, Shah PR, Vanikar AV, Shah VR, Trivedi HL: **High mortality in critically ill patients infected with 2009 pandemic influenza A (H1N1) with pneumonia and acute kidney injury.** *Saudi J Kidney Dis Transpl* 2011, **22:**83-89.
33. Langenegger E, Coetzee A, Jacobs S, Le Roux A, Theron G: **Severe acute respiratory infection with influenza A (H1N1) during pregnancy.** *S Afr Med J* 2009, **99:**713-716.
34. Libster R, Bugna J, Coviello S, Hijano DR, Dunaiewsky M, Reynoso N, Cavalieri ML, Guglielmo MC, Areso MS, Gilligan T, Santucho F, Cabral G, Gregorio GL, Moreno R, Lutz MI, Panigasi AL, Saligari L, Caballero MT, Egues Almeida RM, Gutierrez Meyer ME, Neder MD, Davenport MC, Del Valle MP, Santidrian VS, Mosca G, Garcia Dominguez M, Alvarez L, Landa P, Pota A, Bolonati N, et al: **Pediatric hospitalizations associated with 2009 pandemic influenza A (H1N1) in Argentina.** *N Engl J Med* 2010, **362:**45-55.
35. Liem NT, Tung CV, Hien ND, Hien TT, Chau NQ, Long HT, Hien NT, Mai LQ, Taylor WRJ, Wertheim H, Farrar J, Khang DD, Horby P: **Clinical features of human influenza A (H5N1) infection in Vietnam: 2004-2006.** *Clin Infect Dis* 2009, **48:**1639-1646.
36. Liu C, Schwartz BS, Vallabhaneni S, Nixon M, Chin-Hong PV, Miller SA, Chiu C, Damon L, Lawrence Drew W: **Pandemic (H1N1) 2009 infection in patients with hematologic malignancy.** *Emerg Infect Dis* 2010, **16:**1910-1917.

37. Martin SS, Hollingsworth CL, Norfolk SG, Wolfe CR, Hollingsworth JW: **Reversible cardiac dysfunction associated with pandemic 2009 influenza A(H1N1).** *Chest* 2010, **137:**1195-1197.

38. Martin-Loeches I, Lisboa T, Rhodes A, Moreno RP, Silva E, Sprung C, Chiche JD, Barahona D, Villabon M, Balasini C, Pearse RM, Matos R, Rello J, The ESICM H1N1 Registry Contributors: **Use of early corticosteroid therapy on ICU admission in patients affected by severe pandemic (H1N1)v influenza A infection.** *Intensive Care Med* 2011, **37:**272-283.

39. Miller RR, 3rd, Markewitz BA, Rolfs RT, Brown SM, Dascomb KK, Grissom CK, Friedrichs MD, Mayer J, Hirshberg EL, Conklin J, Paine R, 3rd, Dean NC: **Clinical findings and demographic factors associated with ICU admission in Utah due to novel 2009 influenza A(H1N1) infection.** *Chest* 2010, **137:**752-758.

40. Miroballi Y, Baird JS, Zackai S, Cannon J-M, Messina M, Ravindranath T, Green R, Della-Latta P, Jenkins S, Greenwald BM, Furuya EY, Graham PL, 3rd, Sonnett FM, Platt S, Delamora P, Saiman L: **Novel influenza A(H1N1) in a pediatric health care facility in New York City during the first wave of the 2009 pandemic.** *Arch Pediatr Adolesc Med* 2010, **164:**24-30.

41. Muller MP, McGeer AJ, Hassan K, Marshall J, Christian M: **Evaluation of pneumonia severity and acute physiology scores to predict ICU admission and mortality in patients hospitalized for influenza.** *PLoS ONE [Electronic Resource]* 2010, **25**.

42. Mulrennan S, Tempone SS, Ling ITW, Williams SH, Gan G-C, Murray RJ, Speers DJ: **Pandemic influenza (H1N1) 2009 pneumonia: CURB-65 score for predicting severity and nasopharyngeal sampling for diagnosis are unreliable.** *PLoS ONE [Electronic Resource]* 2010, **5:**e12849.

43. New South Wales public health n: **Progression and impact of the first winter wave of the 2009 pandemic H1N1 influenza in New South Wales, Australia.** *Euro Surveill* 2009, **14**.

44. Nguyen-Van-Tam JS, Openshaw PJM, Hashim A, Gadd EM, Lim WS, Semple MG, Read RC, Taylor BL, Brett SJ, McMenamin J, Enstone JE, Armstrong C, Nicholson KG: **Risk factors for hospitalisation and poor outcome with pandemic A/H1N1 influenza: United Kingdom first wave (May-September 2009).** *Thorax* 2010, **65:**645-651.

45. Orellano PW, Reynoso JI, Carlino O, Uez O: **Protection of trivalent inactivated influenza vaccine against hospitalizations among pandemic influenza A (H1N1) cases in Argentina.** *Vaccine* 2010, **28:**5288-5291.

46. Ormsby CE, de la Rosa-Zamboni D, Vázquez-Pérez J, Ablanedo-Terrazas Y, Vega-Barrientos R, Gómez-Palacio M, Murakami-Ogasawara A, Ibarra-Ávalos JA, Romero-Rodríguez D, Ávila-Ríos S, Reyes-Terán G: **Severe 2009 pandemic influenza A (H1N1) infection and increased mortality in patients with late and advanced HIV disease.** *AIDS* 2011, **25:**435-439.

47. Pereira JM, Moreno R, Matos R, Martin-Loeches I, Cecconi M, Lisboa T, Rhodes A, Rello J: **Curb-65 in pandemic influenza a (H1N1)v: Results of the esicm influenza a (H1N1)v registry.** *Intensive Care Med* 2010, **36:**S370.

48. Riera M, Payeras A, Marcos MA, Viasus D, Farinas MC, Segura F, Torre-Cisneros J, Martin-Quiros A, Rodriguez-Bano J, Vila J, Cordero E, Carratala J: **Clinical presentation and prognosis of the 2009 H1N1 influenza A infection in HIV-1-infected patients: a Spanish multicenter study.** *AIDS* 2010, **24:**2461-2467.

49. Sahoo JN, Banani P, Afzal A, Singh RK, Mohan G, Baronia AK: **Pandemic (H1N1) 2009 influenza: experience from a critical care unit in India.** *Indian J Crit Care Med* 2010, **14:**156-159.

50. Sam IC, Abdul-Murad A, Karunakaran R, Rampal S, Chan Y-F, Nathan AM, Ariffin H: **Clinical features of Malaysian children hospitalized with community-acquired seasonal influenza.** *Int J Infect Dis* 2010, **14 Suppl 3:**e36-40.
51. Sandar S, Chow A, Lye D, Barkham T, Leo YS: **Risk factors associated with severe influenza in tropical Singapore.** *Int J Infect Dis* 2010, **14:**S48.
52. Schnell D, Mayaux J, de Bazelaire C, Legoff J, Feuillet S, Scieux C, Andreu-Gallien J, Darmon M, Baruchel A, Schlemmer B, Azoulay E: **Risk factors for pneumonia in immunocompromised patients with influenza.** *Respir Med* 2010, **104:**1050-1056.
53. Silvennoinen H, Peltola V, Vainionpaa R, Ruuskanen O, Heikkinen T: **Incidence of influenza-related hospitalizations in different age groups of children in Finland: A 16-year study.** *Pediatr Infect Dis J* 2011, **30:**e24-e28.
54. Skarbinski J, Jain S, Bramley A, Lee EJ, Huang J, Kirschke D, Stone A, Wedlake T, Richards SM, Page S, Ragan P, Bullion L, Neises D, Williams RM, Petruccelli BP, Vandermeer M, Lofy KH, Gindler J, Finelli L: **Hospitalized patients with 2009 pandemic influenza A (H1N1) virus infection in the United States--September-October 2009.** *Clin Infect Dis* 2011, **52 Suppl 1:**S50-59.
55. Staikowsky F, D'Andrea C, Filleul L, Guiserix J, Vanhecke C, Winer A, Michault A: **Outbreak of influenza pandemic virus A(H1N1) 2009 infections in Emergency Department, Saint-Pierre, Reunion Island. July-August 2009. [French] Prise en charge dans un service d'urgence de l'epidemie a virus pandemique A(H1N1) 2009 a Saint-Pierre, ile de La Reunion. Juillet-Aout 2009.** *Presse Med* 2010, **39:**e147-e157.
56. Stein M, Tasher D, Glikman D, Shachor-Meyouhas Y, Barkai G, Yochai AB, Leibovitz E, Hausman-Kedem M, Hess A, Megged O, Kassis I, Gresario G, Somekh E: **Hospitalization of children with influenza A(H1N1) virus in Israel during the 2009 outbreak in Israel: a multicenter survey.** *Arch Pediatr Adolesc Med* 2010, **164:**1015-1022.
57. Strouse JJ, Reller ME, Bundy DG, Amoako M, Cancio M, Han RN, Valsamakis A, Casella JF: **Severe pandemic H1N1 and seasonal influenza in children and young adults with sickle cell disease.** *Blood* 2010, **116:**3431-3434.
58. Tutuncu EE, Ozturk B, Gurbuz Y, Haykir A, Sencan I, Kuscu F, Dede G, Kilic AU, Senturk GC: **Clinical characteristics of 74 pandemic H1N1 influenza patients from Turkey. Risk factors for fatality.** *Saudi Med J* 2010, **31:**993-998.
59. van 't Klooster TM, Wielders CC, Donker T, Isken L, Meijer A, van den Wijngaard CC, van der Sande MA, van der Hoek W: **Surveillance of hospitalisations for 2009 pandemic influenza A(H1N1) in the Netherlands, 5 June - 31 December 2009.** *Euro Surveill* 2010, **15:**14.
60. Viasus D, Ramon Paño-Pardo J, Cordero E, Campins A, López-Medrano F, Villoslada A, Fariñas MC, Moreno A, Rodríguez-Baño J, Antonio Oteo J, Martínez-Montauti J, Torre-Cisneros J, Segura F, Carratalà J: **Effect of immunomodulatory therapies in patients with pandemic influenza A (H1N1) 2009 complicated by pneumonia.** *J Infect* 2011, **62:**193-199.
61. Wada K, Nishiura H, Kawana A: **An epidemiological analysis of severe cases of the influenza A (H1N1) 2009 virus infection in Japan.** *Influenza Other Respi Viruses* 2010, **4:**179-186.
62. Xi X, Xu Y, Jiang L, Li A, Duan J, Du B, Chinese Critical Care Clinical Trial G: **Hospitalized adult patients with 2009 influenza A(H1N1) in Beijing, China: risk factors for hospital mortality.** *BMC Infect Dis* 2010, **10:**256.

63. Yang P, Deng Y, Pang X, Shi W, Li X, Tian L, Zhang Y, Wang X, Huang F, Raina MC, Wang Q: **Severe, critical and fatal cases of 2009 H1N1 influenza in China.** *J Infect* 2010, **61:**277-283.

64. Yu H, Gao Z, Feng Z, Shu Y, Xiang N, Zhou L, Huai Y, Feng L, Peng Z, Li Z, Xu C, Li J, Hu C, Li Q, Xu X, Liu X, Liu Z, Xu L, Chen Y, Luo H, Wei L, Zhang X, Xin J, Guo J, Wang Q, Yuan Z, Zhou L, Zhang K, Zhang W, Yang J, et al: **Clinical characteristics of 26 human cases of highly pathogenic avian influenza A (H5N1) virus infection in China.** *PLoS ONE [Electronic Resource]* 2008, **3:**e2985.

65. Zenciroglu A, Kundak AA, Aydin M, Okumus N, Dursun A, Ipek MS, Karagol BS, Hakan N, Karadag NN, Altas AB, Korukluoglu G: **Swine influenza A (H1N1) virus infection in infants.** *Eur J Pediatr* 2011, **170:**333-338.
